# Case ascertainment of a potential centrally-implemented, automated system for national surveillance of healthcare-associated infections, England, 2016 to 2023

**DOI:** 10.2807/1560-7917.ES.2025.30.42.2500066

**Published:** 2025-10-23

**Authors:** Jack Cregan, Olisaeloka Nsonwu, Dimple Chudasama, Susan Hopkins, Berit Muller-Pebody, Russell Hope, Ann Sarah Walker, Thai Phuong Quan

**Affiliations:** 1 Nuffield Department of Medicine, University of Oxford, Oxford, United Kingdom; 2 The National Institute for Health Research Health Protection Research Unit in Healthcare Associated Infections and Antimicrobial Resistance at the University of Oxford, Oxford, United Kingdom; 3 The National Institute for Health Research Oxford Biomedical Research Centre, University of Oxford, Oxford, United Kingdom; 4 Clinical and Public Health Group, UK Health Security Agency, London, United Kingdom

**Keywords:** Healthcare-associated infection, surveillance, data collection, automation

## Abstract

**BACKGROUND:**

Mandatory reporting of healthcare-associated infections (HCAI) in England is conducted locally by acute hospital groups and can be a large burden on healthcare staff.

**AIM:**

We aimed to determine the case ascertainment of a potential centrally-implemented, automated HCAI surveillance system in England using preexisting data feeds at the UK Health Security Agency.

**METHODS:**

We compared monthly case numbers submitted between 1 April 2016 and 31 March 2023 by acute hospital groups (locally-implemented surveillance) to routinely-collected laboratory and hospital encounter records (centrally-implemented surveillance) for all infections under mandatory surveillance in England. Since laboratories can serve multiple hospitals, we compared several methods of assigning laboratory-confirmed cases to hospital groups.

**RESULTS:**

Locally-implemented vs centrally-implemented surveillance identified: meticillin-resistant *Staphylococcus aureus* bacteraemias 5,453 vs 5,859 (ratio 1.07), meticillin-susceptible *S. aureus* bacteraemias 84,680 vs 83,326 (0.98), *Escherichia coli* bacteraemias 281,100 vs 275,133 (0.98), *Klebsiella* species bacteraemias 65,877 vs 67,301 (1.02), *Pseudomonas aeruginosa* bacteraemias 25,862 vs 25,715 (0.99), *Clostridioides difficile* infections (CDI) 94,054 v 90,942 (0. 97) respectively. Assigning hospital groups by linking laboratory records to hospital encounters produced lower monthly mean absolute difference (MAD) vs locally-implemented surveillance than using laboratory records alone. MAD was 0.65 cases/month for bacteraemias, 2.99 for CDI; differences occurred in both directions. MAD decreased over time for bacteraemias but increased from April 2021 onwards for CDI.

**CONCLUSION:**

Centrally-implemented surveillance could be feasible for bacteraemias in England due to comparable case numbers with local surveillance. However, more research is needed around understanding and managing data quality of automated feeds, particularly for CDI.

**Key public health message**

**What did you want to address in this study and why?**

We wanted to investigate if automated data feeds already in place at the UK Health Security Agency (UKHSA) could be used to identify cases of healthcare-associated infections (HCAI) currently subject to mandatory reporting in England. If so, these feeds could potentially be used for a centrally-implemented surveillance system, relieving the burden of reporting from local healthcare staff.

**What have we learnt from this study?**

Monthly HCAI case numbers per hospital group generated centrally at UKHSA using automated data feeds were comparable to those reported by local teams for bacteraemia, at less than one case per month difference on average across all months and hospital groups. Differences were larger for *Clostridioides difficile* infections, at around three cases per month.

**What are the implications of your findings for public health?**

Producing comparable case numbers is a key measure in assessing the equivalence of two different surveillance systems, suggesting a centrally-implemented surveillance approach could potentially be feasible for bacteraemias in England. However, more research is needed around understanding and managing data quality of automated feeds, particularly for *C. difficile* infections.

## Introduction

Healthcare-associated infections (HCAI) are a large burden on hospitals and effective surveillance is essential to understanding and managing their impact. In England, it has been mandatory to report cases of infection by key organisms since 2001. These data are collected locally by each hospital group (organisational units providing local hospital services) before being submitted to a central system where they are reviewed within the national context. While reporting is important for public health, infection control staff inevitably spend considerable time fulfilling reporting requirements [1,2].

Automated surveillance (whether fully automated or only partially) has been implemented successfully in some local settings [3,4] and can reduce staff workload [5]. Unfortunately, automated systems can require significant IT expertise to implement and maintain, which only some local organisations will possess. However, these organisations would still need to satisfy any national reporting requirements, while organisations without local automation would continue to have to collate and submit their information manually.

An alternative approach would be to bypass local teams and implement automated surveillance centrally. However, large-scale and/or national implementations are still rare [6] and the accuracy of case ascertainment is unclear [7]. In England, voluntary surveillance through automated laboratory reporting of positive isolates has been in place nationally for over 40 years, with similar overall numbers and trends in mandatory and voluntary-reported cases varying depending on the pathogen [8]. In this study, we measure case ascertainment across different hospital groups over time as a marker of the feasibility of transitioning from a locally-implemented surveillance approach [6] (where cases are identified locally then submitted to a coordinating centre) to a centrally-implemented surveillance approach (where the coordinating centre identifies cases from automated feeds of local data). Acceptable levels of accuracy are essential here since case numbers are reported publicly for hospital groups.

## Methods

### Case definitions for healthcare-associated infection mandatory surveillance

In England, HCAI mandatory surveillance requires the reporting of all cases of blood cultures positive for *Staphylococcus aureus, Escherichia coli*, *Klebsiella* species and *Pseudomonas aeruginosa*, whether or not the patient attended a hospital [9]. *S. aureus* cases are classified as meticillin-resistant *S. aureus* (MRSA) if resistant to meticillin, cefoxitin, oxacillin or flucloxacillin, or if positive for *mecA* or *mecC;* otherwise, cases are classified as meticillin-susceptible *S. aureus* (MSSA). Additionally, cases of *Clostridioides difficile* infection (CDI) in people aged 2 years or older must be reported. Under current guidelines [10], *C. difficile* testing should be a two-stage process, with the second stage testing for *C. difficile* toxin, and only toxin-positive cases included as CDI.

As instructed in the United Kingdom Health Security Agency (UKHSA) reporting protocol [9], repeat positive tests from the same patient are deduplicated into ‘infection episodes’ or ‘cases’, with a new case declared if a positive specimen is found more than 14 days since the first positive specimen of the episode for bacteraemia, or more than 28 days for CDI.

### Locally-implemented surveillance data

English hospitals are grouped into National Health Service (NHS) trusts, which are organisational units serving a geographical area or specific specialty, with different trusts able to serve overlapping geographical areas. There are currently 211 NHS trusts in England (termed hospital groups in this study), 137 of which provide acute secondary care. There are pathology laboratories located in 96 of the 137 acute hospital groups, and none in non-acute hospital groups. Some of the pathology laboratories test samples from multiple acute hospital groups, and for operational reasons, acute hospital groups can send samples to different laboratories.

Acute hospital groups are responsible for reporting infection episodes occurring in patients in their care when the index specimen is collected (see Supplementary material for a diagram describing organisations responsible for different data submissions). If the index specimen is not collected within an acute hospital group, the acute hospital group in which the testing laboratory is located is responsible for reporting the case. This information is submitted to the UKHSA using a web portal called the HealthCare Associated Infection Data Capture System (DCS).

We downloaded the number of monthly infection episodes per pathogen (*C. difficile,* *E. coli*, *Klebsiella* spp, *P. aeruginosa, MRSA and MSSA*) per reporting hospital group from DCS between April 2016 and March 2023. *Klebsiella* spp. and *P. aeruginosa* have only been under mandatory surveillance since April 2017. We did not include the 19 non-NHS organisations who also reported to DCS (comprising 0.4% of cases).

### Centrally-implemented surveillance data and assigning cases to hospital groups

Data on positive test results for the included pathogens are also routinely submitted from pathology laboratories (109 laboratories between April 2016 and March 2023; 96 in operation in March 2023) to the UKHSA Second Generation Surveillance System (SGSS) (see Supplementary material for a diagram describing organisations responsible for different data submissions). We combined SGSS records of these pathogens isolated from blood (stool for CDI) into infection episodes following the same inclusion criteria as for mandatory HCAI surveillance (see the section on case definitions). *C. difficile* positive tests reported as toxin negative in SGSS were excluded. *S. aureus* episodes were categorised as MRSA if any isolate within the episode was recorded as phenotypically resistant to meticillin, cefoxitin, oxacillin or flucloxacillin, otherwise they were categorised as MSSA. The presence of *mecA* and *mecC* genes could not be determined since they are inconsistently tested by laboratories (depending on local processes) and these data are not collected centrally. Susceptibility testing category ‘I’ (susceptible, increased exposure) was considered as resistant before 1 January 2019 and susceptible afterwards following the change in the European Committee on Antimicrobial Susceptibility Testing definitions [11].

As the pathology laboratories do not have a one-to-one relationship with acute hospital groups, we explored different methods of assigning cases to the appropriate hospital group, either based on the SGSS data alone, or in conjunction with hospital encounter records.

Second Generation Surveillance System records contain multiple data fields that correspond to hospital groups: (i) the laboratory testing the sample (operated by an acute hospital group, denoted ‘laboratory hospital group’), (ii) the location where the specimen was collected (an acute or non-acute hospital site or another location such as a general practice, denoted ‘collection site’) and (iii) the (acute or non-acute) hospital group of the ‘collection site’, if any (denoted ‘recorded hospital group’). As the ‘recorded hospital group’ is known within UKHSA to be unreliable, we developed a heuristic algorithm to create a new ‘amended hospital group’, by selecting the predominant hospital group per collection site based on linked inpatient admissions data from Hospital Episode Statistics (HES) [12] (see Supplementary methods for details on derivation of amended hospital group). This ‘amended hospital group’ differed from the ‘recorded hospital group’ for 474 (26.9%) sites and 85,617 (15.0%) test results. For each infection episode, we used the location of the index specimens only.

**Figure 1:**
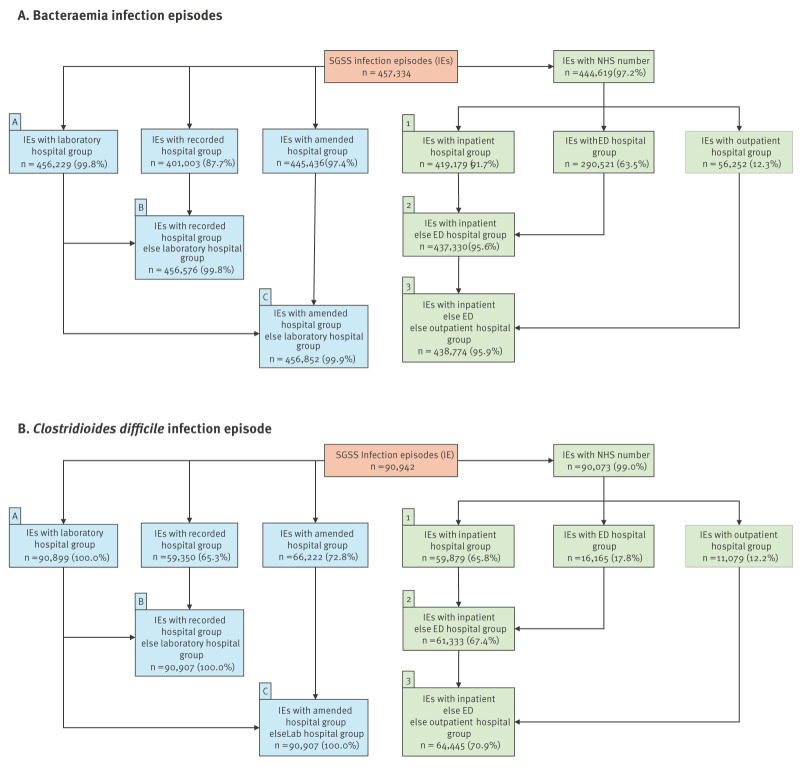
Methods and completeness of assigning an acute hospital group to infection episodes identified from centrally-implemented surveillance for (A) bacteraemias^a^ and (B) *Clostridioides difficile*, England, April 2016–March 2023

We combined the laboratory/recorded/amended hospital groups in different ways to assign infection episodes to acute hospital groups (Figure 1). For Method A, we simply used the ‘laboratory hospital group’. For Methods B and C, we followed the two-step process used for locally-implemented surveillance, firstly finding the acute hospital group of the collection site (if any), by using the ‘recorded hospital group’ (Method B) or the ‘amended hospital group’ (Method C). If the collection site was not at an acute hospital group, we used the 'laboratory hospital group’ instead.

ED: emergency department; IEs: infection episodes; SGSS: Second Generation Surveillance System (routine microbiology laboratory results).

^a^ Includes meticillin-resistant *Staphylococcus aureus*, meticillin-susceptible *S. aureus***,** *Escherichia coli, Klebsiella* species and *Pseudomonas aeruginosa.*

Given the known problems with the ‘recorded hospital group’ field in SGSS, we hypothesised that linking individual laboratory records to contemporaneous hospital encounter records from HES [12,13] would be better than using the SGSS data alone. Therefore, we used NHS numbers to link cases to either (i) inpatient encounters only (Method 1), (ii) inpatient else emergency department (ED) encounters (Method 2), and (iii) inpatient else ED else outpatient encounters (Method 3). As only calendar dates (and not times) are available, where the index specimen collection date was within 1 day either side of an inpatient stay, ED attendance or outpatient appointment, that infection episode was linked to that healthcare encounter. Where more than one acute hospital group was linked to the same index specimen (1.1%, 0.3% and 0.5% of infection episodes when linked to inpatient, ED and outpatient encounters, respectively), the hospital group was selected in preference order of: (i) coincidence of specimen collection date with encounter dates (prioritising earlier admissions for inpatients), (ii) specimen collection date pre-encounter, then (iii) specimen collection date post-encounter (see Supplementary methods for details on linking laboratory records to patient encounters). Of note, while the NHS number is a unique patient identifier, it is sometimes missing (particularly for overseas visitors), mistyped or there may not have been a contemporaneous hospital encounter to link the laboratory record to. Therefore, we combined each of Methods 1–3 with Method C to select a hospital group when one was not found using Methods 1, 2, or 3 alone. These became Methods 1C, 2C and 3C.

### Analysis methods

Using the above-mentioned criteria for reporting cases to DCS, for each of the 137 acute hospital groups we compared the monthly number of cases reported to the current DCS system (locally-implemented surveillance) to the monthly number of cases had surveillance been conducted using SGSS (centrally-implemented surveillance) using the different methods of assigning acute hospital groups. Following DCS practices, hospital groups that had merged or demerged during the study period were recoded to reflect the hospital groups in March 2023.

We visually inspected the quality of the two sets of data using the *daiquiri* R package [14] (see Supplementary methods for details on how this was conducted), checking the time series plots it created for indications of interrupted data feeds. This was done for each reporting hospital group for locally-implemented surveillance data and for each ‘amended hospital group’ for centrally-implemented surveillance data. We excluded hospital group-months that had anomalously low numbers of days with reported cases compared with the preceding or later months from subsequent comparisons. For CDI, more than one third of laboratories reported more than 99% of their *C. difficile* results with an unspecified toxin status. Therefore, records with unknown toxin status (39%) were included in comparisons. See Supplementary material for figures showing examples of anomalous behaviour in the data feeds and for toxin status of *C. difficile* positive tests by laboratory.

To compare case numbers between locally-implemented surveillance vs centrally-implemented surveillance, we calculated monthly mean and mean absolute differences per pathogen and hospital group, since monthly case numbers is the metric that is reported publicly for hospital groups. We then applied two-sided paired t-tests with a significance level of 5%. We repeated this for the different combinations of the centrally-implemented hospital group assignment methods mentioned above, to see if increasing the complexity of the assignment method led to improved accuracy. For the best performing method, we stratified the monthly differences over time and across hospital groups. All analyses were conducted using R version 4.3.0 [15].

## Results

### Total cases by pathogen

**Figure 2:**
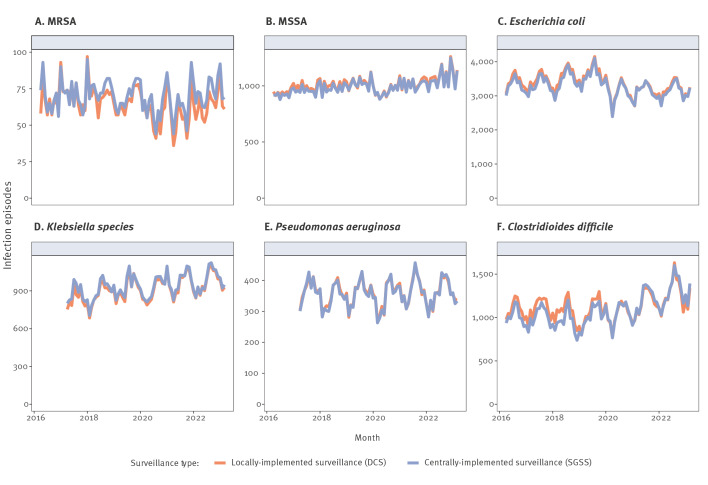
Total bacteraemia and *Clostridioides difficile* infection episodes identified per month from locally-implemented and centrally-implemented surveillance, England, April 2016–March 2023

DCS: HealthCare Associated Infection Data Capture System; MRSA: meticillin-resistant *Staphylococcus aureus*; MSSA: meticillin-susceptible *S. aureus*; SGSS: Second Generation Surveillance System.

Between 1 April 2016 and 31 March 2023, locally-implemented vs centrally-implemented surveillance identified total numbers of: MRSA bacteraemias 5,453 vs 5,859 (ratio: 1.07), MSSA bacteraemias 84,680 vs 83,326 (ratio: 0.98), *E. coli* bacteraemias 281,100 vs 275,133 (ratio: 0.98), *Klebsiella* spp. bacteraemias 65,877 vs 67,301 (ratio: 1.02), *P. aeruginosa* bacteraemias 25,862 vs 25,715 (ratio: 0.99) and CDI 94,054 vs 90,942 (ratio: 0. 97) episodes respectively (Figure 2) (Table 1). Ratios were broadly stable over time, except for MRSA bacteraemia, where the overall excess of cases in centrally-implemented surveillance increased over time and for CDI, where fewer cases were identified from centrally-implemented surveillance before 2020, with more cases thereafter.

**Table 1:** Total number of bacteraemia and *Clostridioides difficile* infection episodes identified from locally-implemented surveillance (DCS) and centrally-implemented surveillance (SGSS) by pathogen, England, April 2016–March 2023

**Year**	**MRSA^a^**			**MSSA**			***Escherichia coli***			***Klebsiella* species**		
	**SGSS**	**DCS**	**Ratio**	**SGSS**	**DCS**	**Ratio**	**SGSS**	**DCS**	**Ratio**	**SGSS**	**DCS**	**Ratio**
2016–17	850	825	1.03	11,169	11,467	0.97	39,255	40,522	0.97	NA	NA	NA
2017–18	851	849	1.00	11,604	11,912	0.97	39,655	40,980	0.97	10,220	9,772	1.05
2018–19	868	807	1.08	11,779	12,062	0.98	41,958	43,127	0.97	10,954	10,695	1.02
2019–20	856	815	1.05	12,133	12,200	0.99	42,418	43,234	0.98	11,240	11,047	1.02
2020–21	764	696	1.10	11,626	11,666	1.00	36,346	36,645	0.99	11,340	11,144	1.02
2021–22	787	674	1.17	12,073	12,263	0.98	37,364	37,878	0.99	11,567	11,403	1.01
2022–23	883	787	1.12	12,942	13,110	0.99	38,137	38,714	0.99	11,980	11,816	1.01
**Total**	**5,859**	**5,453**	**1.07**	**83,326**	**84,680**	**0.98**	**275,133**	**281,100**	**0.98**	**67,301**	**65,877**	**1.02**
**Year**	***Pseudomonas aeruginosa***			**All bacteraemia**			***Clostridioides difficile*** **(toxin positive plus unknown toxin status)**			***C. difficile*** **(toxin positive only)**		
	**SGSS**	**DCS**	**Ratio**	**SGSS**	**DCS**	**Ratio**	**SGSS**	**DCS**	**Ratio**	**SGSS**	**DCS**	**Ratio**
2016–17	NA	NA	NA	51,274	52,814	0.97	11,952	12,847	0.93	7,666	12,847	0.60
2017–18	4,291	4,303	1.00	66,621	67,816	0.98	12,076	13,282	0.91	7,945	13,282	0.60
2018–19	4,119	4,183	0.98	69,678	70,874	0.98	11,221	12,287	0.91	7,303	12,287	0.59
2019–20	4,300	4,343	0.99	70,947	71,639	0.99	12,658	13,223	0.96	7,953	13,223	0.60
2020–21	4,248	4,288	0.99	64,324	64,439	1.00	12,499	12,500	1.00	7,632	12,500	0.61
2021–22	4,347	4,336	1.00	66,138	66,554	0.99	14,543	14,289	1.02	8,960	14,289	0.63
2022–23	4,410	4,409	1.00	68,352	68,836	0.99	15,993	15,626	1.02	8,897	15,626	0.57
**Total**	**25,715**	**25,862**	**0.99**	**457,334**	**462,972**	**0.99**	**90,942**	**94,054**	**0.97**	**56,356**	**94,054**	**0.60**

DCS: Health Care Associated Infection Data Capture System; MRSA: meticillin-resistant *Staphylococcus aureus*; MSSA: meticillin-susceptible *S. aureus*; NA: not applicable; SGSS: Second Generation Surveillance System.

^a^ The excess in MRSA cases obtained from SGSS was largely attributable to a single hospital group, which had 382 episodes in SGSS vs 21 in DCS. Excluding this outlier results in a total ratio of 1.01 but does not alter the increasing trend over time.

### Comparison of hospital group assignment methods

Assigning an acute hospital group to each infection episode using centrally-implemented surveillance was not straightforward, and completeness varied by method of assignment (Figure 1). Almost all infection episodes could be assigned a ‘laboratory hospital group’ (> 99.8%, Method A), whereas only 91.7% of bacteraemia and 65.8% of CDI episodes could be assigned a hospital group by linking to inpatient admissions (Method 1).

Assessment of data quality demonstrated clear challenges with missing data before 2020–21 for centrally-implemented surveillance (e.g. due to failure of automated data feeds). This led to 1.5–5.2% and 6.6–12.5% of acute hospital group-months being excluded from subsequent analyses for bacteraemias and CDI, respectively, dropping to 0.0–1.5% and 5.2–8.1% after 2020–21 (see Supplementary material for monthly percentage of acute hospital groups from centrally-implemented surveillance with data quality issues, and for details on how the data quality inspection was conducted). There were no obvious data quality issues with locally-implemented surveillance data.

Monthly infection episode numbers per acute hospital group were compared in the remaining 24,520 hospital group-months between locally-implemented surveillance and across different methods of assigning acute hospital groups in centrally-implemented surveillance (see Supplementary material for comparison of monthly numbers per acute hospital group, assignment method and pathogen). For bacteraemias, using the ‘amended hospital group’ (Method C) produced smaller mean and mean absolute differences vs locally-implemented surveillance compared to Methods A and B that used only laboratory data (mean difference Method C: -0.0109, mean difference Method A: -0.0224 (p = 0.714 Method C vs Method A), mean difference Method B: -0.0285 (p = 0.354 Method C vs Method B)) (Table 2). Linking specific bacteraemia episodes incrementally to inpatient, ED and outpatient encounters (Methods 1, 2, 3), successively reduced differences to locally-implemented surveillance numbers. Adding a hospital group obtained from laboratory-reported data where no hospital encounter was linked (Methods 1C, 2C and 3C) had similar low mean and mean absolute differences compared to locally-implemented surveillance. Method 3C produced the lowest mean difference compared to locally-implemented surveillance (mean difference: -0.00929, p = 0.226), but there was little difference compared to Method C (mean difference: 0.0016). Method 1C produced the lowest mean absolute difference (mean absolute difference: 0.6502) compared to locally-implemented surveillance.

**Table 2:** Differences between monthly number of infection episodes per acute hospital group identified from locally-implemented surveillance (DCS) and centrally-implemented surveillance (SGSS), for bacteraemias and *Clostridioides difficile,* England, April 2016–March 2023

Hospital group assignment method (Figure 1)	Description	Total cases assigned to acute hospital group	Difference in total cases assigned vs DCS	Mean absolute difference of monthly assigned cases vs DCS	Mean difference of monthly assigned cases vs DCS^a^		p value^a^	Comparator method	Mean absolute difference of monthly assigned cases vs comparator	Mean difference of monthly assigned cases vs comparator^a^		p value^a^
					Mean difference	95% CI				Mean difference	95% CI	
**All bacteraemia**												
Reference method	Locally-implemented surveillance (DCS)	454,556	NA	NA	NA		NA	NA	NA	NA		NA
A	Laboratory hospital group	453,363	-1,193	3.0520	-0.0224	-0.0859 to 0.0411	0.489	NA	NA	NA		NA
B	Recorded hospital group else A	453,036	-1,520	2.0316	-0.0285	-0.0706 to 0.0134	0.182	vs A	2.9363	-0.0061	-0.068 to 0.0561	0.846
C	Amended hospital group else A	453,975	-581	0.7538	-0.0109	-0.0290 to 0.0071	0.236	vs A vs B	2.6648	0.0115	-0.0501 to 0.0731	0.714
									1.4784	0.0177	-0.0197 to 0.0550	0.354
1	Inpatient hospital group	416,552	-38,004	1.0162	-0.7151	-0.7352 to 0.6950	< 0.001	NA	NA	NA		NA
2	Inpatient else ED hospital group	434,613	-19,943	1.0061	-0.6994	-0.7194 to 0.6794	< 0.001	vs 1	0.0158	0.0158	0.0142 to 0.01727	< 0.001
3	Inpatient else ED else outpatient hospital group	436,046	-18,510	0.9660	-0.6377	-0.6572 to 0.6182	< 0.001	vs 2	0.0617	0.0617	0.0592 to 0.06418	< 0.001
1C^b^	1 else C	454,059	-497	0.6502	-0.0093	-0.0244 to 0.0057	0.223	vs B vs C	1.6709	0.0192	-0.0194 to 0.0578	0.329
									0.2775	0.0016	-0.0086 to 0.0117	0.761
2C^b^	2 else C	454,061	-495	0.6503	-0.0094	-0.0244 to 0.0057	0.222	vs 1C	0.0003	0.0000	-0.0002 to 0.0001	0.796
3C^b^	3 else C	454,062	-494	0.6572	-0.0093	-0.0244 to 0.0058	0.226	Vs C vs 2C	0.2940	0.0016	-0.0087 to 0.0119	0.759
									0.0218	0.0000	-0.0013 to 0.0014	0.933
***C. difficile***												
Reference method	Locally-implemented surveillance (DCS)	86,060	NA	NA	NA		NA	NA	NA	NA		NA
A	Laboratory hospital group	89,701	3,641	4.4316	0.3468	0.2046 to 0.4890	< 0.001	NA	NA	NA		NA
B	Recorded hospital group else A	89,141	3,081	3.6187	0.2935	0.1772 to 0.4097	< 0.001	vs A	2.1419	-0.0533	-0.1395 to 0.0328	0.225
C	Amended hospital group else A	89,436	3,376	3.0582	0.3216	0.2121 to 0.4310	< 0.001	vs A vs B	1.8970	-0.0252	-0.1069 to 0.0564	0.545
									1.0091	0.0281	-0.0244 to 0.0806	0.295
1	Inpatient hospital group	58,910	-27,150	3.6421	-2.5860	-2.6734 to 2.4985	< 0.001	NA	NA	NA		NA
2	Inpatient else ED hospital group	58,988	-27,072	3.6381	-2.5785	-2.6659 to 2.4912	< 0.001	vs 1	0.0074	0.0074	0.0056 to 0.0093	< 0.001
3	Inpatient else ED else outpatient hospital group	62,222	-23,838	3.5142	-2.2705	-2.3588 to 2.1822	< 0.001	vs 2	0.3080	0.3080	0.2953 to 0.3208	< 0.001
1C^b^	1 else C	89,457	3,397	3.0067	0.3236	0.2148, 0.4323	< 0.001	vs B vs C	1.1138	0.0301	-0.0233 to 0.0834	0.269
									0.1533	0.0020	-0.0070 to 0.0110	0.665
2C^b^	2 else C	89,457	3,397	3.0063	0.3236	0.2148 to 0.4323	< 0.001	vs 1C	0.0008	0.0000	-0.0005 to 0.0005	1.000
3C^b^	3 else C	89,426	3,366	2.9936	0.3206	0.2124 to 0.4288	< 0.001	vs C vs 2C	0.2610	-0.0010	-0.0131 to 0.0112	0.878
									0.1224	-0.0030	-0.0104 to 0.0045	0.435

CI: confidence interval; DCS: HealthCare Associated Infection Data Capture System; ED: emergency department; NA: not applicable.

^a^ Two-sided paired t-test on monthly case numbers per pathogen per acute hospital group.

^b^ Combination of Methods 1, 2, or 3 with Method C from Figure 1.

Excluding laboratory/hospital-months with substantial data quality issues, see Methods and Supplementary material.

For CDI, mean and mean absolute differences in monthly case numbers between centrally-implemented vs locally-implemented surveillance were larger than those for bacteraemia, with no assignment methods producing comparable case numbers to locally-implemented surveillance (Table 2). Method 3C produced both the lowest mean difference (0.3206) and the lowest mean absolute difference (2.9936) in case numbers when comparing to locally-implemented surveillance; again, the difference in number of cases per month using Method 3C vs Method C was very small (mean difference: -0.0010, mean absolute difference: 0.2610). Differences in performance between the various assignment methods were broadly similar to those for bacteraemia.

### Variation over time and between hospital groups

**Figure 3:**
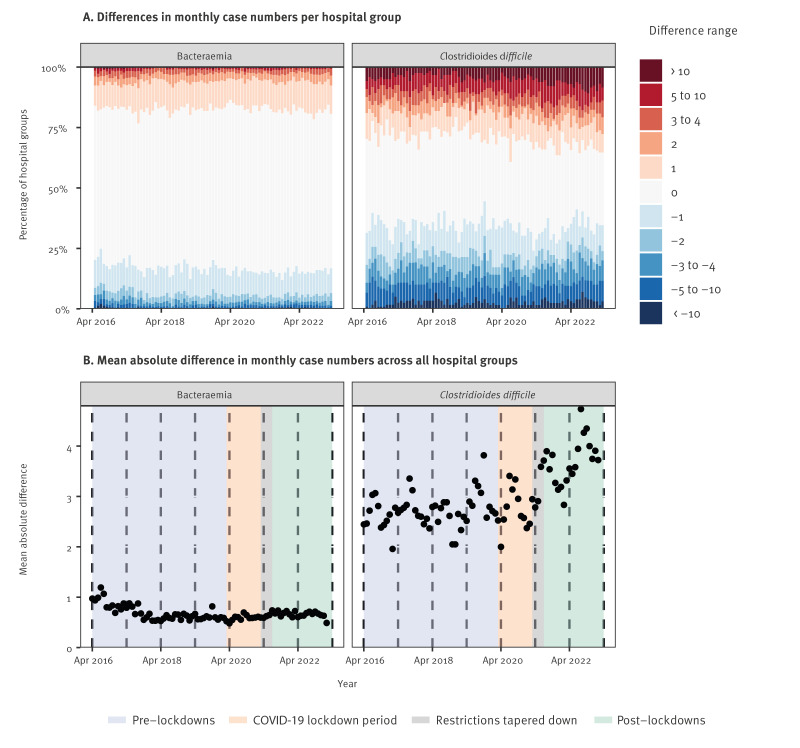
(A) differences in monthly case numbers per hospital group and (B) mean absolute difference in monthly case numbers across all hospital groups for bacteraemia and *Clostridioides difficile* infection episodes using centrally-implemented surveillance Method 3C vs locally-implemented surveillance, England, April 2016–March 2023

To investigate changes in case ascertainment over time, we compared monthly case numbers between locally-implemented surveillance and the best-performing assignment method for centrally-implemented surveillance (Method 3C) per acute hospital group. For bacteraemias, the mean absolute difference across all hospital groups decreased from around one case per hospital group in April 2016 to around 0.7 cases per hospital group each month from April 2018 onwards (Figure 3). Although small, differences occurred in both directions i.e. sometimes slightly more bacteraemias were identified from centrally-implemented than locally-implemented surveillance, and vice versa. The percentage of hospital group-months with zero difference (i.e. where centrally- and locally-implemented surveillance produced the same number of cases) increased slightly from 64.9% (between April 2016 and March 2018), to 66.1% (between April 2018 and March 2020) and thereafter to 67.8%. Patterns were broadly similar for individual pathogens (see Supplementary material for differences over time per pathogen), although the magnitude of differences aligned with total case numbers.

In contrast, for CDI mean absolute difference between centrally-implemented vs locally-implemented surveillance across all hospital groups increased from around 2.5 cases per hospital group in April 2016 to around 4 cases per hospital group in March 2023 (Figure 3). This increase coincided with an increase in the proportion of positive CDI tests from centrally-implemented surveillance with toxin status classified as unknown (see Supplementary material for toxin status of *C. difficile* positive tests over time). The percentage of hospital group-months with zero difference between centrally- and locally-implemented surveillance cases correspondingly decreased slightly from 37.6% between April 2016 and May 2021 to 33.5% between June 2021 and March 2023.

**Figure 4:**
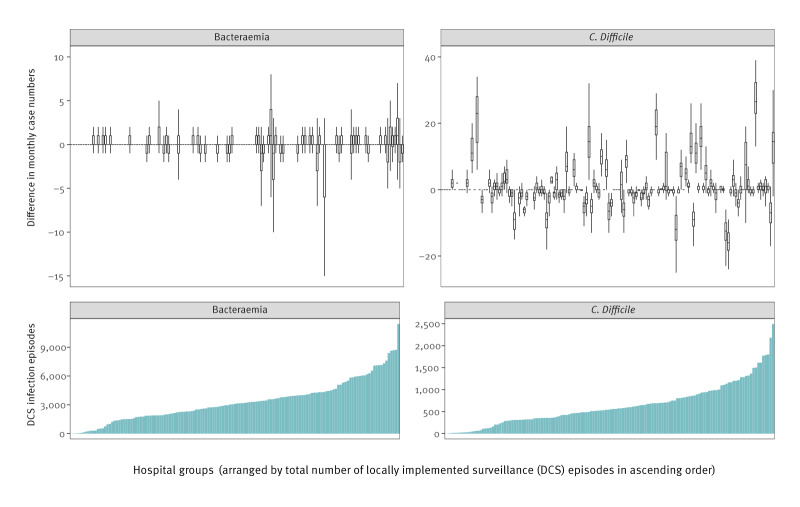
Distribution of differences in monthly case numbers for acute hospital groups using centrally-implemented surveillance method 3C vs locally-implemented surveillance for bacteraemias and *Clostridioides difficile* infection, ordered by total number of cases reported to locally-implemented surveillance England, April 2016–March 2023

To investigate variation across different hospital groups further, we restricted to the three most recent financial years (1 April 2020 to 31 March 2023), where mean absolute differences in monthly case numbers between centrally- and locally-implemented surveillance were relatively stable for bacteraemias (mean of 0.61 cases per month), but less stable and somewhat higher (mean of 3.34 cases per month) for CDI (Figure 3). For bacteraemias, while no hospital groups had zero differences across *all* months, 84 of 137 (61%) had zero differences at both the 25th and 75th percentiles (Figure 4). Differences were slightly larger among hospital groups with higher numbers of cases. Overall, the absolute difference was ≤ 1 case in 21,888 of 24,520 (89.3%) hospital group-months in this period (≤ 2 cases in 23,245/24,520 hospital group-months, 94.8%). In contrast, for CDI, only 25/134 (19%) hospital groups (remaining in analysis after data quality exclusions) had zero differences at both the 25th and 75th percentiles, while 115 of 134 (86%) hospital groups had a more than five cases difference at both 25th and 75th percentile (Figure 4). In contrast to bacteraemias, hospital groups with larger differences tended to consistently have more cases in one direction or the other (17/134 hospital groups with 25th percentile > 2, 14/134 with 75th percentile < -2).

DCS: HealthCare Associated Infection Data Capture System.

Different y-axis scales on each plot; outliers excluded from box-and-whisker diagrams.

## Discussion

In this study we have shown that centrally-implemented surveillance can produce comparable case numbers to locally-implemented surveillance for bacteraemias in England. The larger discrepancies in CDI cases require more investigation, but appear to be related to difficulties that the current automated feeds have in determining toxin status, particularly given the two-stage testing process. Our comparisons also demonstrate that these discrepancies are concentrated at particular hospital pathology laboratories, rather than being system-wide, so may be best reduced by targeting resources at those laboratories. While the proportion of CDI cases that could be linked to hospital encounters was considerably lower than for bacteraemias, one can reasonably expect proportionately more genuine community CDI cases. Therefore, our use of the ‘laboratory hospital group’ where no hospital encounter is found will likely be correctly assigning these. Differences in case ascertainment between CDI and bacteraemia highlight that – while it is tempting to assume that centrally-implemented surveillance is a straightforward approach given the systematic and automated method of data acquisition – in practice, there may be numerous complexities that require careful attention.

For this initial feasibility study, we focussed on a comparison of monthly case numbers at acute hospital groups. While it is important to be able to obtain similar case numbers from centrally-implemented vs locally-implemented surveillance, this alone is not a sufficient measure for evaluating surveillance methods; timeliness and the completeness/accuracy of relevant epidemiological information also need to be investigated. While we have shown that linkage between laboratory data and national administrative datasets is good, with more than 95% of bacteraemia episodes linking to contemporaneous hospital encounters, it is not yet known how well these administrative data would provide the additional data points needed to satisfy reporting requirements and/or understand changes in epidemiology. Nevertheless, there is still a trade-off in terms of the local burden of collecting such data and identifying where its value may still justify its capture locally.

One interesting aspect is determining which data source is ‘correct’. In an ideal world, there would be an independent ‘ground truth’ of all HCAI against which both locally-implemented and centrally-implemented surveillance could be compared. However, this does not exist, and historically, public health bodies have relied on mandatory surveillance schemes or extrapolations of point prevalence surveys to estimate HCAI burden. Given the complexities in reporting, it is difficult to be confident that the current locally-implemented surveillance system is necessarily the gold standard. Reasons why cases might be missed with the current system include high workloads and challenges identifying locations of, and times between, repeat isolates of the pathogens under surveillance. In contrast, provided that all positive isolations are received from microbiology laboratories, algorithmic approaches to defining infection episodes and assigning a responsible acute hospital group can be uniformly applied. The key point is that any centrally-implemented system should be fair and transparent.

Automated data feeds are not always as reliable as might be expected; our analysis of data quality demonstrated that frequent (and ideally daily) monitoring is essential in order to identify faults as they happen and reduce missing data. Additional investment in IT infrastructure and support, both centrally and at participating laboratories, may be required to maintain uptime. Laboratory data received at UKHSA currently comprises positive isolations plus antimicrobial susceptibility testing results. Receipt of all tests conducted, whether positive or negative, would make it easier to identify data quality issues using automated methods such as statistical process control [16], particularly for smaller laboratories and rarer infections. As these data were missing, we were unable to use these methods to assess data quality in our study. As more laboratories move to molecular susceptibility testing which is currently rare in England, mechanisms to capture for example, the presence of genes such as *mecA/C* will be required, otherwise MRSA cases will be undercounted. These mechanisms will also be needed to maintain the accuracy of SGSS for other reporting purposes, and so are part of the ongoing maintenance that any automated system needs beyond its initial inception to ensure it keeps up with any changes in the source data. Any resources saved at local hospitals will also therefore need to be balanced against any increased IT costs.

Identifying the hospital group responsible for case reporting was not as straightforward as originally expected, since the data come from laboratories rather than the hospital groups themselves. We found that triangulation with hospital encounter data improved this task, and that data fields that purportedly contain the desired value (e.g. the ‘recorded hospital group’), cannot automatically be relied upon. Accuracy is important since case numbers are reported publicly for the hospital groups to which they are assigned. The lack of a straightforward one-to-one mapping could be argued to be a weakness of a centrally-implemented approach. However, this is an inherent limitation to be overcome by any system that involves reconciling information that exists in multiple systems and can be reported differently, whether this is automated, semi-automated or manual. All automated systems require some level of data standardisation and a linkage strategy to deal with incomplete, inaccurate or inconsistently reported data, which is what we investigated in the present study. However, this is not unique to a centrally-implemented approach: a locally-implemented approach would still need to resolve these types of issues before reporting the same information to the coordinating centre, e.g. by checking that the case has not also been reported by another hospital. Despite protocols and training, it is not clear that local staff will inevitably make better decisions. The benefit of a centrally-implemented automated system is consistent application of rules for resolving discrepancies and the reduced burden for acute hospital groups who currently only have a manual or partially-automated approach to data capture. One important challenge, however, is timeliness. Not only in terms of time taken for data to be submitted centrally [17], but also that there may be potentially slower local responses if outbreaks are not recognised in real-time due to the need for locally-implemented surveillance and reporting being removed.

We found unexpected differences in case numbers for individual pathogens. For MRSA bacteraemia, we identified more cases and more over time with the centrally-implemented approach. The excess was largely attributable to a single hospital group suggesting that data quality was likely a contributing factor, perhaps with screening samples being included erroneously. However, this trend remained after excluding that particular hospital group. For CDI, the reasons for the shift over time in ratio of cases identified in centrally-implemented vs locally-implemented surveillance are unclear, but may be partly explained by contemporaneous increases in cases with unknown toxin status. Again, this highlights the need for continual monitoring of data feeds.

We are aware of only one national automated HCAI surveillance system in operation in Europe. This is perhaps not surprising, since up until 2018, only 50% of European Union/ European Economic Area countries were able to receive automated data feeds from their clinical laboratories, with only Denmark receiving data from all their laboratories [18]. Launched in 2015, Denmark’s automated system was found to have a sensitivity of only 36% when compared with point prevalence surveys [7], but the system was still considered useful given its ability to observe changes in an individual hospital’s data over time.

## Conclusion

In England, between 2016 and 2023, the number of healthcare-associated bacteraemia identified using automated data feeds was comparable to the number found by local surveillance. This suggests that a centrally-implemented surveillance system could potentially be feasible. However, more research is needed around understanding and managing data quality of automated feeds, particularly for CDI. Large-scale centrally-implemented surveillance is still new territory and there are many lessons to be learned.

## Data Availability

The data underlying this article were accessed from UKHSA. Monthly locally-implemented surveillance data are publicly available as national statistics and are downloadable from https://www.gov.uk/search/research-and-statistics?parent=/health-and-social-care/health-protection-services-health-surveillance-and-reporting-programmes&content_store_document_type=statistics_published&topic=7d55894e-5ba5-4495-8962-e7650e9a0559&organisations%5B%5D=uk-health-security-agency. The other datasets used are not publicly available as they contain patient-level data, but can be requested from the respective data controllers. Data from HES are available on request from NHS England via https://digital.nhs.uk/services/data-access-request-service-dars. Data from SGSS can be requested from UKHSA via https://www.gov.uk/government/publications/accessing-ukhsa-protected-data.

## Supplementary Data

1567000 Supplementary Material
