# Supplementary material for: Case ascertainment of a potential centrally-implemented, automated system for national surveillance of healthcare-associated infections, England, 2016 to 2023
Source: Euro Surveill. 2025 Oct 23;30(42):2500066. doi: 10.2807/1560-7917.ES.2025.30.42.2500066 (PMC12555115; doi:10.2807/1560-7917.ES.2025.30.42.2500066)
Supplement: Supplementary Material [file 25-00066_QUAN_Supplement.pdf]

This supplementary material is hosted by *Eurosurveillance* as supporting information alongside the article 'Case ascertainment of a potential centrally-implemented, automated system for national surveillance of healthcare-associated infections, England 2016 to 2023', on behalf of the authors, who remain responsible for the accuracy and appropriateness of the content. The same standards for ethics, copyright, attributions and permissions as for the article apply. Supplements are not edited by *Eurosurveillance* and the journal is not responsible for the maintenance of any links or email addresses provided therein.

Supplementary methods

Centrally-implemented surveillance data

Derivation of amended hospital group

For each SGSS laboratory record, there is a data field containing the geographic location where the specimen was collected, typically a specific hospital site or a general practice (denoted collection site). When the collection site is a hospital, there is a further data field which should contain the hospital group that runs the site (denoted recorded hospital group), however it is known that this recorded hospital group is incorrect in a sizeable minority of cases. We therefore developed a heuristic algorithm to find the "correct" hospital group for each collection site, assuming that if the vast majority of records labelled with a particular collection site were taken from patients known to be concurrently admitted at a single hospital group, then that hospital group is likely to be the "correct" one.. Using Hospital Episode Statistics (HES) data obtained from NHS England, we linked each SGSS test result by NHS number to all inpatient admissions where the specimen collection date was between the admission and discharge date inclusive. Across 1,759 reported specimen collection sites, 1,139 were linked to at least one acute or non-acute hospital group. For each of these collection sites, the percentage of (linked) tests that linked to the most commonly linked hospital group ranged from 23-100% (IQR 98-100%), with most collection sites (59%) linked to the same hospital group for all their linked test results. For the 1,032 collection sites that had >85% test results linked to the same hospital group, we assigned that hospital group as the amended hospital group (i.e. setting the cut-off where the percentage of tests linked to a collection site's most commonly linked hospital group tailed off, **Figure S2**). For all other collection sites, the original laboratory recorded hospital group was assigned as the amended hospital group. The amended hospital group differed from the recorded hospital group for 474 (26.9%) sites and 85,617 (15.0%) test results.

Analysis methods

Data quality

Data from both locally- and centrally-implemented surveillance data streams were assessed by visual inspection to identify any clear data quality issues. Data from centrally-implemented surveillance was assessed separately for bacteraemia and CDI.

For each hospital group and data stream, the number of days in each month with a reported bacteraemia/CDI was calculated and plotted over time, using the *daiquiri* R package. For months with suspiciously low numbers of days with reported cases compared to preceding/later months (examples in **Figure S3a**), the relevant months for that hospital group were removed from subsequent analyses as they likely reflected missed data feeds (**Figure S3b**).

Linking laboratory records to patient encounters

For methods **1**, **2**, and **3** in **Figure 1,** we used NHS number to link the laboratory record to contemporaneous inpatient admissions, emergency department attendances and outpatient appointments at acute hospital groups. As only calendar dates (and not times) are available for hospital encounters, where the index specimen collection date was within 1 day either side of an inpatient stay, emergency department attendance or outpatient appointment, that infection episode was linked to that healthcare encounter. Where there were >1 acute hospital groups linked to the same index specimen (in 1.1%, 0.3%, and 0.5% of infection episodes when linked to inpatient, ED, and outpatient encounters respectively), for inpatient linkage we used the hospital group of the earliest (by admission date then by discharge date) admission with admission date ≤ specimen collection date < discharge date; then = discharge date; then = admission date - 1; then = discharge date + 1. Similarly for ED and outpatient linkage, we used the encounter where specimen collection date = attendance/appointment date; then = attendance/appointment date - 1; then = attendance/appointment date + 1. Where there were ties (i.e. two encounters in different acute hospital groups with the same admission and discharge dates or appointment/attendance dates), the earliest in the alphabet by organisation code was chosen (46 inpatient admissions, 382 ED attendances, 589 outpatient appointments).

<u>**Supplementary figures**</u>

Supplementary Figure S1. Responsibility for reporting of mandatory surveillance data and routine microbiological data in England

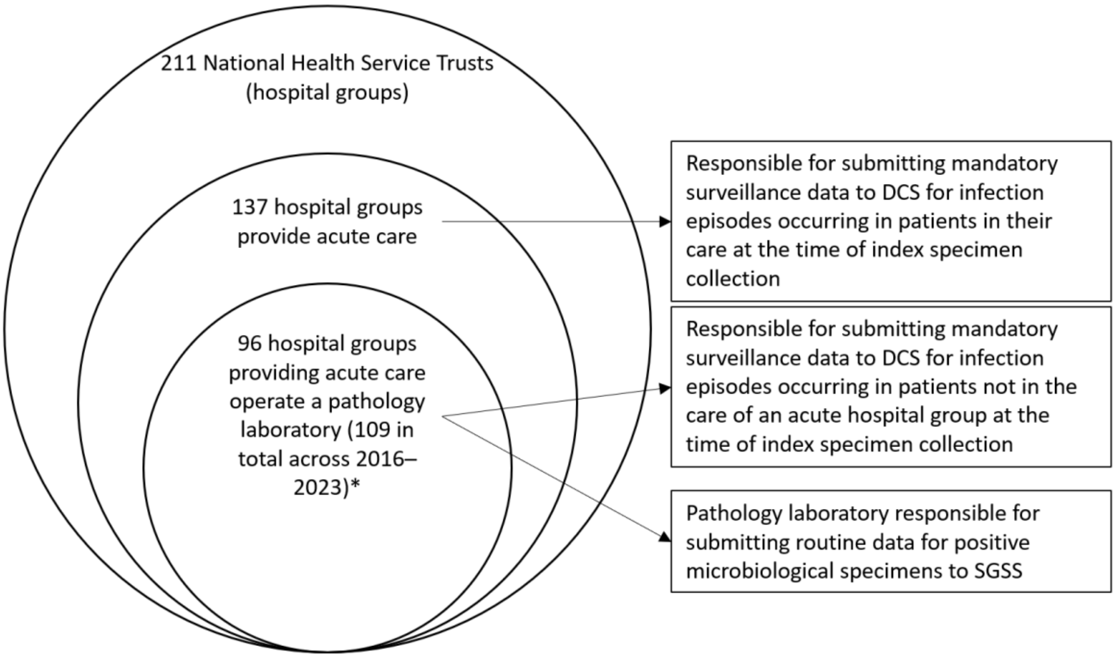

* no pathology laboratories in non-acute hospital groups: some serve multiple acute hospital groups.
Note: DCS=Healthcare Associated Infection Data Capture System. SGSS=Second Generation Surveillance System

Supplementary Figure S2. Across 1,139 SGSS specimen collection sites with a test linked to at least one inpatient admission, the number of (A) collection sites and (B) tests linked to that collection site's most commonly linked hospital group

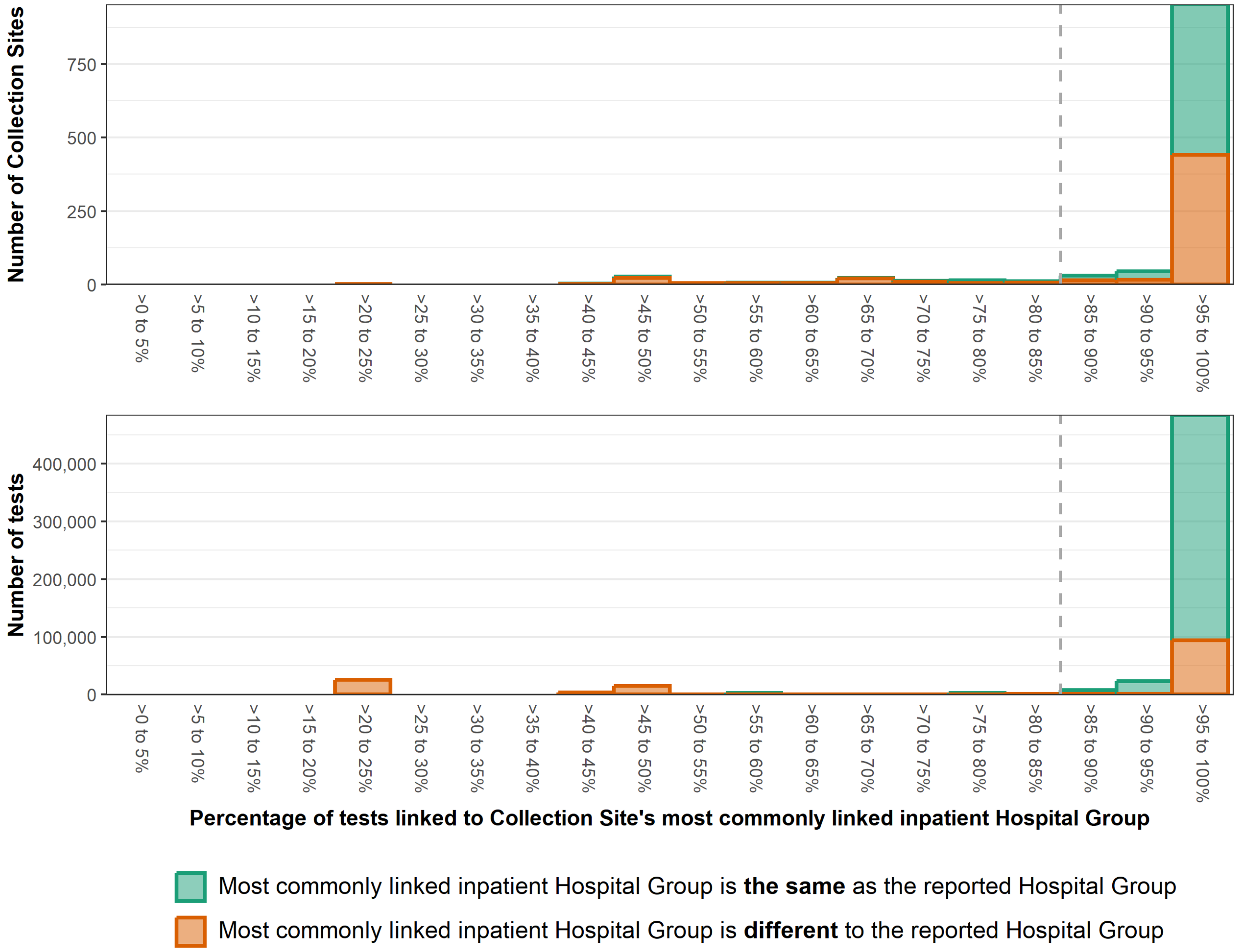

Supplementary Figure S3a. Examples of poor data quality in centrally-implemented surveillance data streams. Each panel contains data for an individual hospital group, and each point shows the number of days in that month with a reported bacteraemia. Note: Panels are based on amended hospital group, see **Figure 1**.

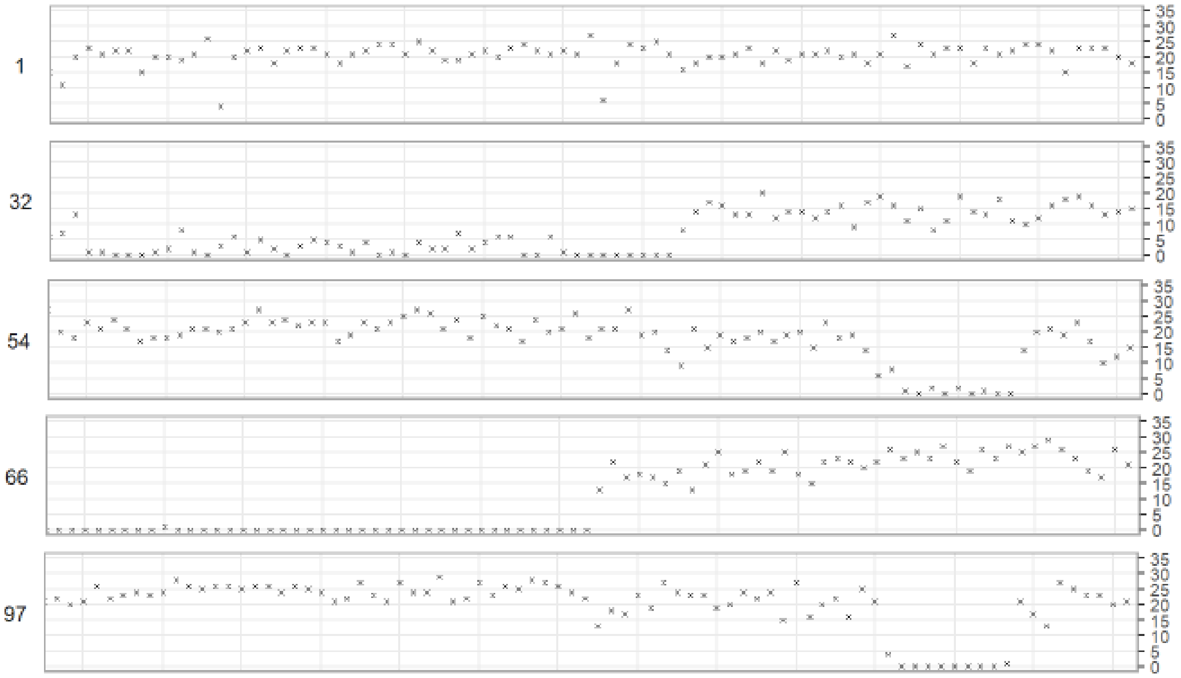

Supplementary Figure S3b. Monthly percentage of acute hospital groups from centrally-implemented surveillance with poor data quality and subsequently excluded from comparisons

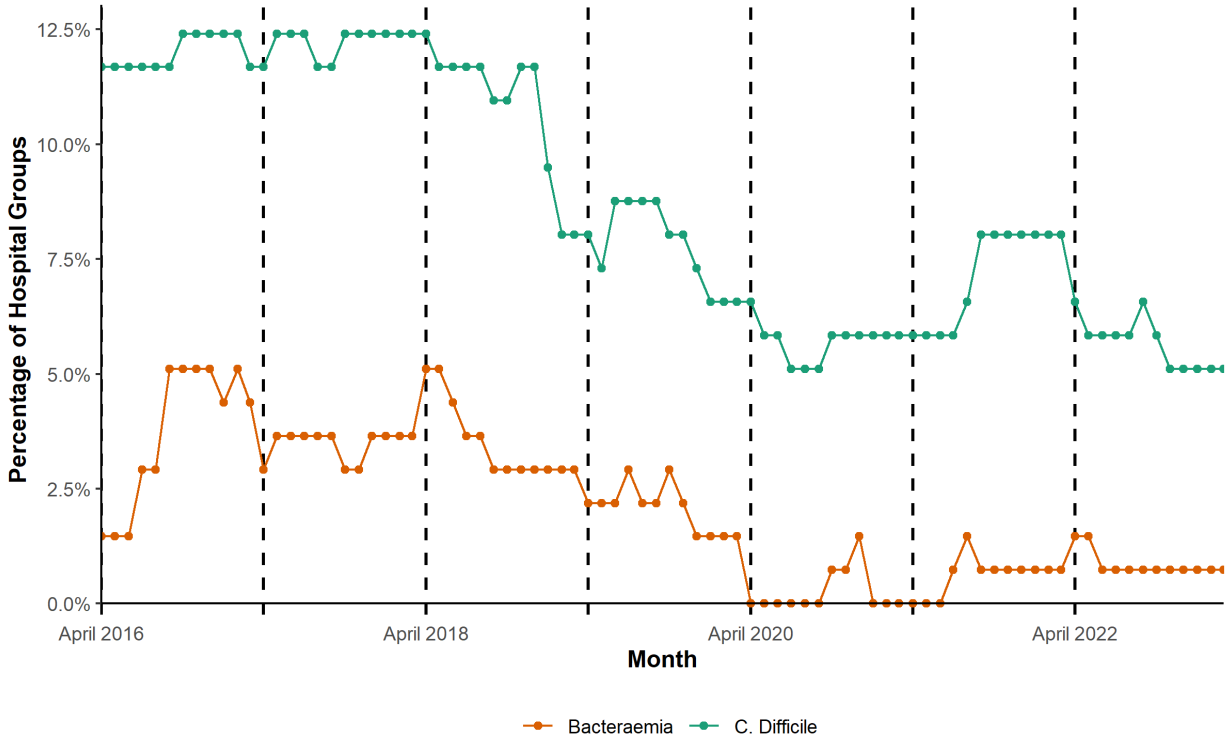

Supplementary Figure S4. Toxin status of *C. difficile* positive tests from centrally-implemented surveillance (SGSS) March 2016 to April 2023

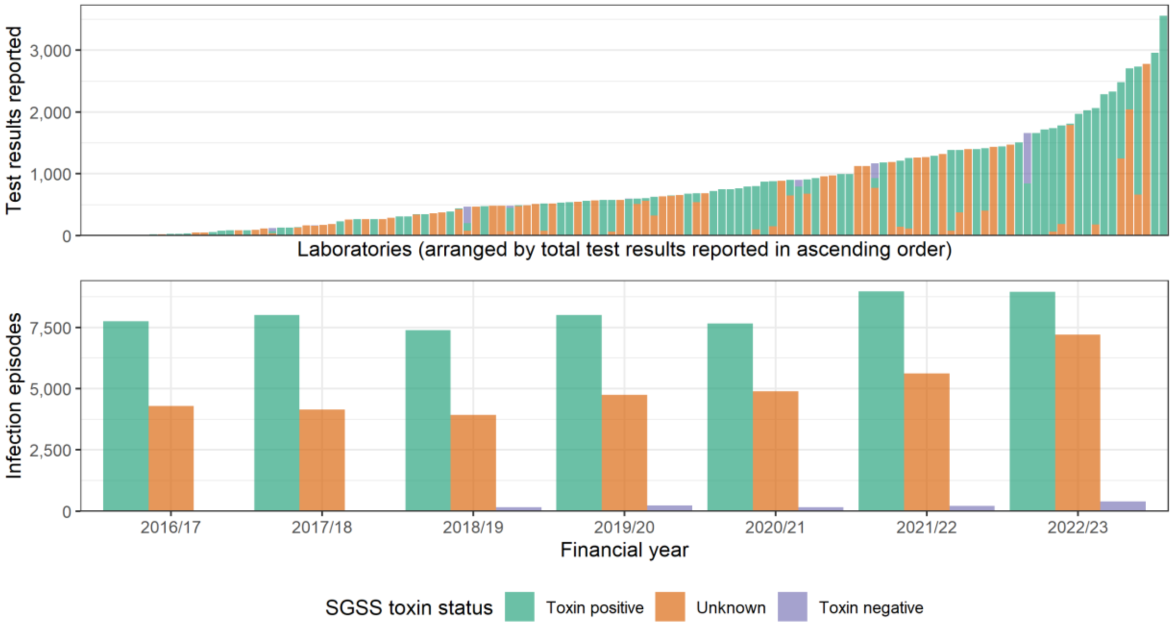

Note: toxin negative test results are shown here for completeness but excluded from all other aspects of the analysis

Supplementary Figure S5. Monthly case numbers per acute hospital group identified from locally-implemented surveillance and different methods of assigning acute hospital group in centrally-implemented surveillance April 2016 to March 2023

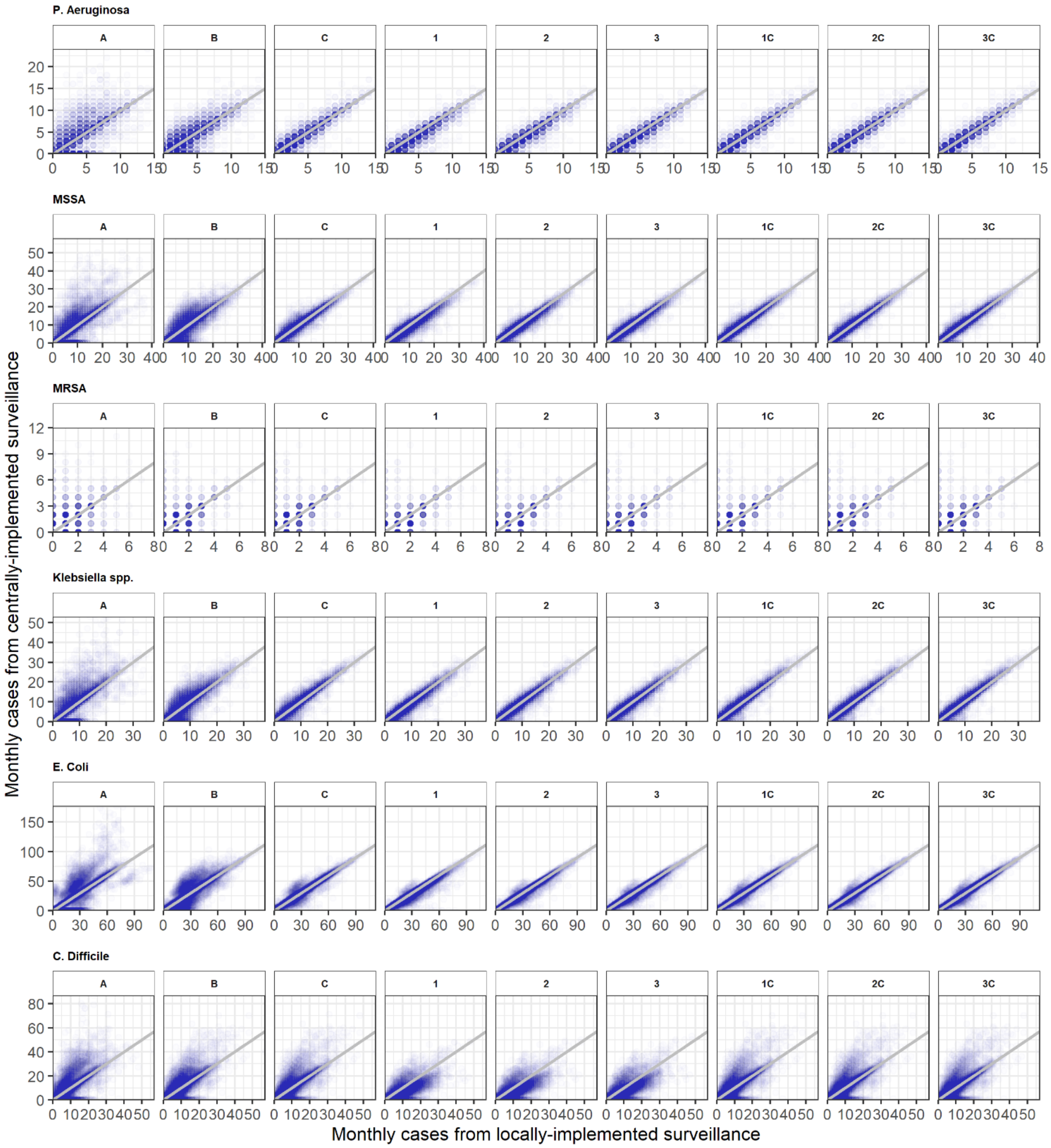

Note: see **Figure 1** for description of methods of assigning acute hospital groups to infection episodes in centrally-implemented surveillance and **Tables 2 and 3** for summary statistics for each comparison.

Supplementary Figure S6. Differences in monthly bacteraemia case numbers per hospital group (top row) and mean absolute difference in monthly bacteraemia case numbers across all hospital groups (bottom row) between centrally-implemented surveillance method 3C vs locally-implemented surveillance, by pathogen

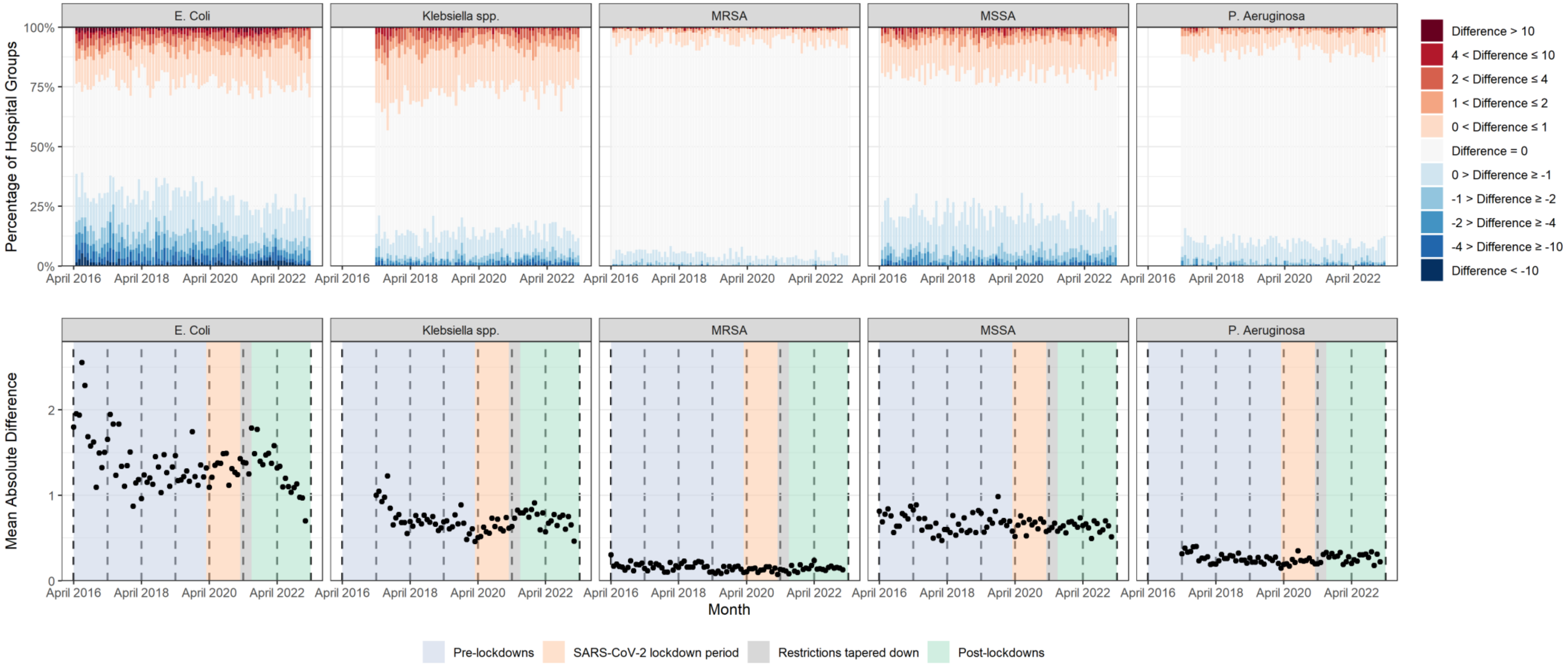

Note: difference is centrally-implemented minus locally-implemented surveillance. See **Figure 4** for difference and mean absolute difference across all bacteraemias and for *C. difficile*
